# Predictors of exclusive breastfeeding among infants under-six months in rural Ethiopia

**DOI:** 10.1371/journal.pone.0341654

**Published:** 2026-02-02

**Authors:** Halefom Shwaye Hantal, Gebrie Melese Abite, Kassahun Animut Metkie

**Affiliations:** Department of Statistics, College of Natural and Computational Sciences, Dilla University, Dilla, Ethiopia; Menzies School of Health Research: Charles Darwin University, AUSTRALIA

## Abstract

**Background:**

Exclusive breastfeeding (EBF) is the practice of providing infants with only breast milk for the first six months of their life, except for medically prescribed drugs or supplements. Globally, EBF prevalence varies with low awareness in developing countries including Ethiopia. Hence, this study identified the significant predictors of the duration of EBF among infants fewer than six months in rural Ethiopia.

**Methods:**

A survival analysis was used to identify the significant predictors of EBF in rural Ethiopia using the 2019 Ethiopia Mini Demographic and Health Survey (EMDHS) dataset.

**Results:**

During the survey period in rural Ethiopia, a significant proportion of mothers (15.1%) do not exclusively breastfeed their infants, which still represents a major health problem. The final multivariable Weibull parametric survival model identified significant predictors of the duration of EBF in rural Ethiopia, such as, mothers with radio access (HR = 1.274, p-value 0.020), mothers delivered by C-section (HR = 0.573, p-value <0.001), mothers residing in Amhara (HR = 0.406, p-value = 0.025), Oromia (HR = 0.379, p-value = 0.017), Somali (HR = 0.112, p-value < 0.001), SNNPR (HR = 0.296, p-value = 0.002), Gambela (HR = 0.285, p-value = 0.002), Harari (HR = 0.220, p-value < 0.001), and Dire Dawa (HR = 0.234, p-value < 0.001), EIBF immediately within the first hour (HR = 1.554, p-value < 0.001), infants aged 0–1 year (HR = 0.226, p-value < 0.001), birth interval of 24 months and more (HR = 0.819, p-value = 0.034), and mothers who were currently married (HR = 0.514, p-value = 0.016).

**Conclusion:**

The duration of EBF among infants less than six months in rural Ethiopia is significantly influenced by household access to radio, cesarean delivery, region, early initiation of breastfeeding, child’s age, preceding birth interval, and marital status at the 5% significance level. To improve EBF practices, there must be targeted interventions that are relevant to regions and better support for 15.1% of mothers.

## Background

Breast milk provides all the essential nutrients children needs for the first 6 months of life [1]. Exclusive breastfeeding (EBF) refers to the act of providing infants with only breast milk for the first six months of their life [2], except for medically prescribed drugs or supplements [3]. It’s recommended that exclusive breastfeeding is required for the first 6 months, meaning children don’t need any other food or drinks other than breast milk during this time [1]. According to the World Health Organization (WHO), exclusive breastfeeding is the best choice for babies under six months. It’s not only cost-effective, but it also dramatically lowers the chances of babies getting diarrhea, malnutrition, and even serious illnesses that can be fatal. Moreover, breastfeeding offers protection against breast cancer for nursing mothers, encourages healthy intervals between pregnancies, and might also reduce their chances of developing ovarian cancer and type 2 diabetes [3]. Feeding complementary foods within the first 6 months will have the adverse effect of reducing breast milk output because the production and release of breast milk are modulated by the frequency and intensity of suckling [1].

Globally, the average rate of exclusive breastfeeding (EBF) from 2015 to 2021 was 48%.As per meta-analysis conducted across 29 countries in sub-Saharan Africa revealed that the prevalence of exclusive breastfeeding varied, with the lowest prevalence recorded at 23.70% in Central Africa and the highest at 56.57% in Southern Africa [4]. In the West Africa region, the prevalence of exclusive breastfeeding (EBF) was 35% from 2015 to 2021, making it one of the lowest rates globally for that period [5,6]. A systematic review in East Africa found that 42% of mothers preferred to exclusively breastfeed their infants for the first six months, while 55.9% of them actually practiced EBF for at least six months [3].

In Ethiopia where the study conducted, according to the 2016 Ethiopian Demographic and Health Survey (EDHS), 58% of infants under six months of age were exclusively breastfed [4]. Furthermore, the 2019 Ethiopian Demographic and Health Survey (EMDHS) indicated that 40% of children were not exclusively breastfed [7]. A systematic review of 16 studies, along with a meta-analysis and two multilevel findings from Ethiopia, indicated that the prevalence of exclusive breastfeeding (EBF) in the country is 59.3%, 59.76%, and 46.8% respectively [8].

Several studies have been conducted in Ethiopia to identify the predictors of exclusive breastfeeding. Some of the key identified predictors are mothers age [2,4,9–12], mothers educational level [2,7,9,10,13–17], family size [9,15,18], region [2,4,10,16,19–22], religion [4,19], residence [2,7,9,14,15,17,19,23], child age [2,14,16,20,21,24], child sex [4,16,21], type of birth [4,10,24], number of living children [2], media access [2], counsel on breastfeeding [11,17,23,24], marital status [2,13,14,8,25], wealth index [2,8,9,13,14,16,20,21], early initiation of breastfeeding [16,17,24,26], caesarean delivery [10,15,26], place of delivery [9,10,14,15,27], ANC visits [4,9,10,12,15,21,25], baby PNC checkup [10,11,14,24]. However, to our knowledge, no prior research in Ethiopia has examined the association between the explanatory variables and time to cessation of EBF among infants less than six months exclusively focused on rural communities at the regional level. Furthermore, previous studies in Ethiopia didn’t investigate some key factors influencing the duration of EBF, such as literacy, birth attendant, birth assistance, mothers’ relationship to the household head, current contraceptive method, and baby formula. This study makes a unique contribution by specifically targeting a population disproportionately affected by low EBF rates. It also offers a comprehensive model comparison, ultimately employing a Weibull parametric survival model to investigate the relationship between the explanatory variables and survival time of infants under six months from the EIBF start date until EBF cessation in months, alongside comparing survivor and hazard functions, taking into account various demographic, socioeconomic, maternal, and health-related factors, by utilizing the most recent nationally representative 2019 EMDHS dataset. Identifying these predictors is important for making focused policies and interventions that can raise EBF rates and, in the end, improve the health of babies in rural Ethiopia. The findings of this study would provide valuable insights to healthcare providers, policymakers, and community organizations that are interested in promoting EBF through customized educational programs and support systems that factor in the unique challenges that mothers in rural areas encounter.

## Methods

### Study setting and data source

This study used secondary data from the children’s data set of the 2019 Ethiopia Mini Demographic and Health Survey (EMDHS). The 2019 EMDHS is a nationwide survey with a nationally representative sample of 9,150 selected households. It is the second Mini Demographic and Health Survey conducted in Ethiopia. The first Ethiopia Mini-DHS, or EMDHS, was conducted in 2014. The 2019 EMDHS provides valuable information on trends in key demographic and health indicators over time. The primary objective of the 2019 EMDHS is to provide up-to-date estimates of key demographic and health indicators.

The sampling frame for the 2019 Ethiopia mini demographic and health survey was a complete list of the 149,093 enumeration areas (EAs), which was performed by the Central Statistical Agency (CSA). An EA is a geographic area that covers an average of 131 households. Administratively, Ethiopia was divided into 11 geographical regions. Each region is subdivided into zones, each zone into woredas, each woreda into towns, and each town into kebeles. The sample for the 2019 EMDHS was designed to provide estimates of key indicators for the country as a whole, for urban and rural areas separately, and for each of the nine regions and the two administrative cities. The 2019 EMDHS sample was stratified and selected in two stages. Each region was stratified into urban and rural areas, yielding 21 sampling strata. Samples of EAs were selected independently in each stratum in two stages.

In the first stage, a total of 305 EAs (93 in urban areas and 212 in rural areas) were selected with probability proportional to EA size (based on the 2019 EPHC frame) and with independent selection in each sampling stratum. In the second stage of selection, a fixed number of 30 households per cluster were selected with an equal probability systematic selection from the newly created household listing. The survey included 9,150 households, and data was collected from 8,885 women of reproductive age (15–49). Of the total 8,885 women, 2,951 from urban and 5,934 from rural households successfully completed the interview. A total of 5753 eligible mothers of under-five children were interviewed about the duration of EBF in the 2019 EMDHS. Of those, data for 1628 mothers were extracted with complete information interviewed for their kid’s duration of EBF.

### Inclusion and exclusion criteria

The study exclusively focused on the children of women born in the last 5 years (0–59 months) who were actively breastfeeding for their six-month duration residing in rural Ethiopia, who were part of a survey at CSA and had EAs with geographical coordinates. On the other hand, the study did not include children who had ever been breastfed, those who had never been breastfed, those not currently being breastfed at the time of the survey, children aged above 5 years old, children from EAs without geographical coordinates, and children residing in urban Ethiopia.

### Study variables

#### Response variable.

The response variable of this study was time to cessation of EBF in months. It was measured by: (1) the mothers were asked about their children’s exclusive breastfeeding (EBF) status if they were given anything other than breast milk in the 24 hours before the interview, for infants aged <6 months. (2) For infants aged >=6 months, the mothers were reflecting on the past: what was their children’s breastfeeding experience during the initial six months of life? Thus, mothers who exclusively breastfed their kids to <6 months were considered as an “event, coded as 1” and “censored, coded as 0” otherwise.

#### Predictor variables.

The considered predictors of the duration of exclusive breastfeeding were socioeconomic, demographic, maternal, and health-related characteristics. Region, religion, household has radio, household has television, family size, literacy, number of children aged 5 years and under resident in the household, household wealth index, currently pregnant, delivery by caesarean section, last birth a caesarean section, birth attendant, birth assistance, antenatal care, number of antenatal visits during pregnancy, total children ever born, current marital status of mothers at the time of the survey, age of household head, mothers age in 5-year groups, highest educational level of the mothers, sex of the household head, mothers relationship to the household head, current contraceptive method, type of birth, sex of child, early initiation of breastfeeding, current age of child, preceding birth interval, birth order, place of delivery, age of mothers at first birth, births in the last five years, counsel on breastfeeding, baby postnatal check, gave child baby formula were considered as the predictors of the duration of exclusive breastfeeding among 5 and under aged children in rural Ethiopia [1].

### Data extraction

The 2019 EMDHS survey datasets were downloaded and processed with permission from the Measure DHS (http://www.dhsprogram.com). The socioeconomic, demographic, maternal, and health-related characteristics and time to cessation of EBF indicator variables were extracted from the children datasets kids recode. The data were weighted for unequal selections among strata. Additional data recoding took place following an in-depth analysis of the detailed datasets and coding.

### Statistical analysis

The statistical analysis of infants’ EBF data was conducted utilizing R version 4.5.0, employing both descriptive statistics (frequency and percentage) and inferential statistics (non-parametric methods, Cox PH model, and parametric survival models) to ascertain the findings. The Kaplan-Meier and Nelson-Aalen survival/hazard curves with the log-rank test were used to compare the survival/hazard of EBF using significant predictor variables. The effect of each predictor variable on the survival time of EBF among infants less than six months in rural Ethiopia was assessed using semi-parametric Cox and parametric survival models. Variables with p-value < 0.2 in bivariate Cox regression and parametric survival models were further analyzed using multivariable Cox regression and parametric survival models. The adjusted hazard ratio (AHR) with their 95% CI and p-value < 0.05 was used to identify the significant predictor variables influencing the survival time of EBF infants under six months in rural Ethiopia.

### Nonparametric methods

An initial step in the analysis of a set of survival data is to present numerical or graphical summaries of the survival time for individuals for a particular group. These summaries can be valuable on their own or serve as a foundation for a more comprehensive analysis of the data. Survival data are conveniently summarized through estimates of the survival function and hazard function. This study discussed methods for estimating these functions from a single sample of survival data, specifically the Kaplan-Meier and Nelson-Aalen estimates [28,29].

#### Kaplan-Meier estimate of survival function.


$$\hat{\text{S}}(\text{t})=\prod_{\text{j}=\text{1}}^{\text{k}}\frac{{\text{n}}_{\text{j}}-{\text{d}}_{\text{j}}}{{\text{n}}_{\text{j}}}$$

Where, ${\text{n}}_{\text{j}}$ is the number of subjects at risk at time $\;{\text{t}}_{\text{j}}$, and ${\text{d}}_{\text{j}}$ is the number of individuals who fail at that time.

#### Nelson-Aalen estimate of survival function.


$$\tilde{\text{S}}(\text{t})=\prod_{\text{j}=\text{1}}^{\text{k}}\text{exp}(\frac{-{\text{d}}_{\text{j}}}{{\text{n}}_{\text{j}}})$$

Where, ${\text{n}}_{\text{j}}$ is the number of subjects at risk at time $\;{\text{t}}_{\text{j}}$, and ${\text{d}}_{\text{j}}$ is the number of individuals who fail at that time.

#### Kaplan-Meier estimate of hazard function.


$$\hat{\text{h}}(\text{t})=\frac{{\text{d}}_{\text{j}}}{{\text{n}}_{\text{j}}\tau_{\text{j}}}$$

Where, ${\text{n}}_{\text{j}}$ is the number of subjects at risk at time $\;{\text{t}}_{\text{j}}$, and ${\text{d}}_{\text{j}}$ is the number of individuals who fail at that time, $\tau_{\text{j}}\;$is the length of the j^th^ time interval.

#### Nelson-Aalen estimate of cumulative hazard function.


$$\tilde{\text{H}}(\text{t})=\sum_{\text{j}=\text{1}}^{\text{k}}\frac{{\text{d}}_{\text{j}}}{{\text{n}}_{\text{j}}}$$

Where, ${\text{n}}_{\text{j}}$ is the number of subjects at risk at time $\;{\text{t}}_{\text{j}}$, and ${\text{d}}_{\text{j}}$ is the number of individuals who fail at that time.

This study utilized survival analysis to identify the significant predictors of exclusive breastfeeding duration among children under five years old in rural Ethiopia. It compared Cox proportional hazards model along with parametric survival models.

### Semi-parametric proportional hazards model

Semi-parametric or Cox proportional hazards model is one of the survival models used to assess the relationship of explanatory variables to survival time.


$$\text{h}(\text{t},\text{X})={\text{h}}_{\text{0}}(\text{t})\text{exp}\left(\sum_{\text{i}=\text{1}}^{\text{35}}\beta_{\text{i}}{\text{X}}_{\text{i}}\right),$$

Where, $\text{X}=({\text{X}}_{\text{1}},\;{\text{X}}_{\text{2}},\;\ldots ,\;{\text{X}}_{\text{35}})$ are the 35 independent variables (predictors) considered in this study in rural Ethiopia. An important feature of this formula, which concerns the proportional hazards (PH) assumption, is that the baseline hazard is a function of t, but does not involve the X’s. In contrast, the exponential expression, involves the X’s, but does not involve t. The X’s here are called time independent X’s. The method of maximum likelihood estimation was used to estimate Cox proportional hazards regression model parameters. The formula for the Cox proportional hazards regression model likelihood function is actually called a “partial” likelihood function rather than a (complete) likelihood function [30].

### Parametric survival models

Let T denote a continuous non-negative random variable representing survival time, with probability density function (pdf) f(t) and cumulative distribution function (cdf) F(t) = Pr{T ≤ t}. We focus on the survival function S(t) = Pr{T > t}, the probability of being alive at t, and the hazard function λ(t) = f(t)/S(t). Let Λ(t) = $\int_{\text{0}}^{t}\lambda (\text{u}textrmdu\;$ denote the cumulative hazard and recall that


S(t) = exp{−Λ(t)}

This study employed some parametric survival distributions and corresponding hazard functions.

#### 1. Exponential distribution.

The exponential distribution has constant hazard over time, i.e., λ(t) = λ ∀t. Thus, the survivor function is S(t) = exp{−λt} and the density is f(t) = λ exp{−λt}.

#### 2. Weibull distribution.

T is Weibull with parameters λ and p, denoted T ∼ W (λ, p), if ${\text{T}}^{\text{p}}$ ∼ E(λ). The cumulative hazard is Λ(t) = ${\left(\lambda \text{t}\right)}^{\text{P}}$, the survivor function is S(t) = exp{−${\left(\lambda \text{t}\right)}^{\text{P}}$}, and the hazard is


$$\lambda (\text{t})=\lambda^{\text{p}}{\text{pt}}^{\text{p}-\text{1}}.$$

#### 3. Log-logistic distribution.

T has a log-logistic distribution with survivor function $\text{S}(\text{t})=\frac{\text{1}}{\text{1}+{\left(\lambda \text{t}\right)}^{\text{p}}}$ and hazard function $\lambda (\text{t})=\frac{{\lambda \text{p}(\lambda \text{t})}^{\text{p}-\text{1}}}{\text{1}+(\lambda \text{t}textrmp}$.

#### 4. Log-normal distribution.

A random variable T has a lognormal distribution if log T has a normal distribution.

In this study, the AIC, BIC, and log likelihood were used to compare the suitability of competing models. After fitting the best fitted survival time model using the statistically significant predictors, the researchers were assessed the significance of the individual parameters and the overall model fit to the EBF kids’ data.

### Ethics statement

This study is a secondary analysis of publicly available data from the 2019 Ethiopia Mini Demographic and Health Survey (EMDHS). The research adheres to international and national ethical standards. Ethical clearance was obtained from the Measure DHS International Program, and direct participant consent is not required due to the secondary nature of the data. The EMDHS data was collected ethically, with participants providing informed consent during data collection. The data’s public availability and use for research align with the original consent obtained from participants.

## Results

**Fig 1:**
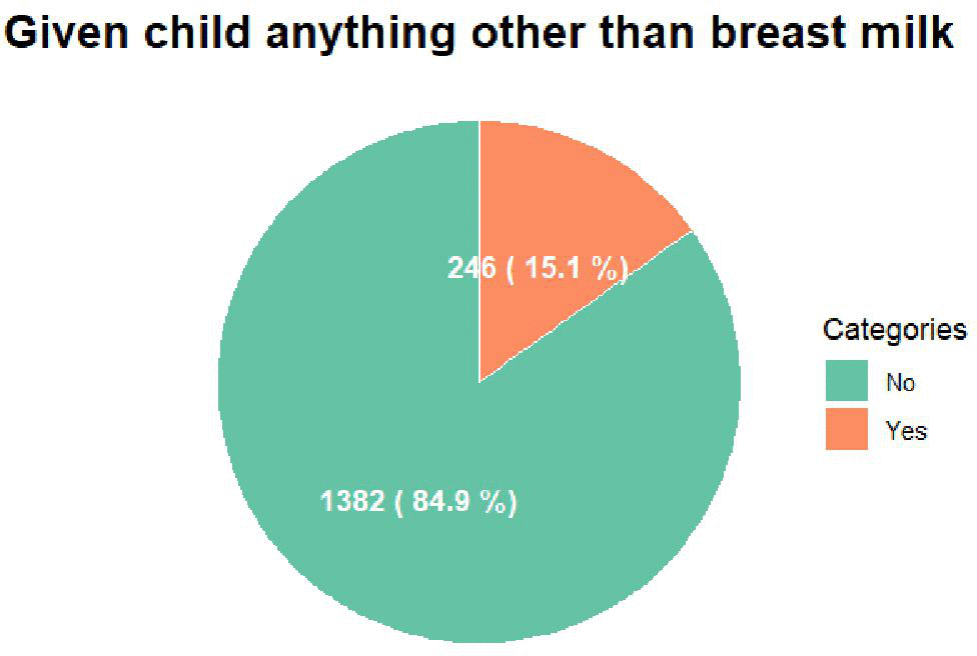
The prevalence of exclusive breastfeeding in rural Ethiopia.

At the time of the survey, although EBF appears relatively high in rural Ethiopia (84.9%), a significant proportion of mothers (15.1%) still discontinue EBF before the recommended 6 months (Fig 1).

The findings of this study revealed that there are significant regional disparities in EBF practices. The highest proportion of children given something other than breast milk (not exclusively breastfeed) is observed in Somali (4.2%) and Afar (2.6%), indicating a pressing concern in these regions. Conversely, regions such as Benishangul (0.55%) exhibit the lowest rates of non-exclusively breastfeed (children given anything other than breast milk), suggesting better adherence to EBF recommendations (Table 1).

**Table 1:** Percentage distribution of EBF by regions of residence, EMDHS 2019 (n = 1628).

Region	Given child anything other than breast milk			Log-rank test
	Yes (%)	No (%)	Total (%)	p-value
Tigray	2 (0.12)	144 (8.85)	146 (8.97)	<0.001
Afar	43 (2.6)	110 (6.8)	153 (9.40)	
Amhara	26 (1.6)	222 (13.6)	248 (15.2)	
Oromia	18 (1.11)	226 (13.88)	244 (15.0)	
Somali	69 (4.2)	66 (4.1)	135 (8.3)	
Benishangul	9 (0.55)	179 (11.0)	188 (11.55)	
SNNPR	34 (2.09)	198 (12.16)	242 (14.25)	
Gambela	21 (1.29)	126 (7.74)	147 (9.03)	
Harari	13 (0.80)	55 (3.38)	68 (4.18)	
Dire Dawa	11 (0.68)	56 (3.44)	67 (4.12)	

### Background characteristics of respondents

Other sociodemographic characteristics of respondents are summarized in Table 2.

**Table 2:** The significant sociodemographic, maternal, and health-related characteristics of respondents, Ethiopia Mini-DHS 2019 (n = 1628).

Variables	Categories	EBF		Log-rank test
		Yes (%)	No (%)	p-value
Household has radio access	No	212 (13.022)	1076 (66.093)	0.006
	Yes	34 (2.088)	306 (18.796)	
C-section delivery	No	222 (13.636)	1328 (81.572)	0.006
	Yes	24 (0.147)	54 (0.332)	
When child put to breast	Immediately/within the first 1 hour	129 (7.924)	310 (19.042)	<0.001
	After 1 hour	117 (7.187)	1072 (65.848)	
Current age of child in years	Above 3 years	3 (0.184)	24 (1.474)	<0.001
	0-1 year	200 (12.285)	1045 (64.189)	
	2-3 years	43 (2.641)	313 (19.226)	
Preceding birth interval (months)	<24 months	44 (2.703)	209 (12.838)	0.08
	>=24 months	202 (12.408)	1173 (72.051)	
Current marital status	Never married/formerly married	4 (0.246)	73 (4.484)	0.008
	Currently married	242 (14.865)	1309 (80.405)	

Survival time, representing the duration of EBF in months, is higher among censored cases; the mean survival time is 15.98 months, with a median of 14 months and a standard deviation of 11.607 months, suggesting a relatively longer duration of EBF with a considerable variability than event/EBF-stopped cases. Other numeric characteristics are summarized in Table 3.

**Table 3:** Summary statistics for continuous variables.

Event	Variable	Mean	Median	Standard deviation
EBF censored	Survival time	15.980	14	11.607
	Number of children 5 and under in household	1.724	2	0.728
	Age of household head	38.045	35	13.407
EBF Stopped/event	Survival time	13.520	11	10.335
	Number of children 5 and under in household	1.923	2	0.875
	Age of household head	36.134	35	11.430
Total	Survival time	15.609	13	11.455
	Number of children 5 and under in household	1.754	2	0.755
	Age of household head	37.756	35	13.141

**Fig 2:**
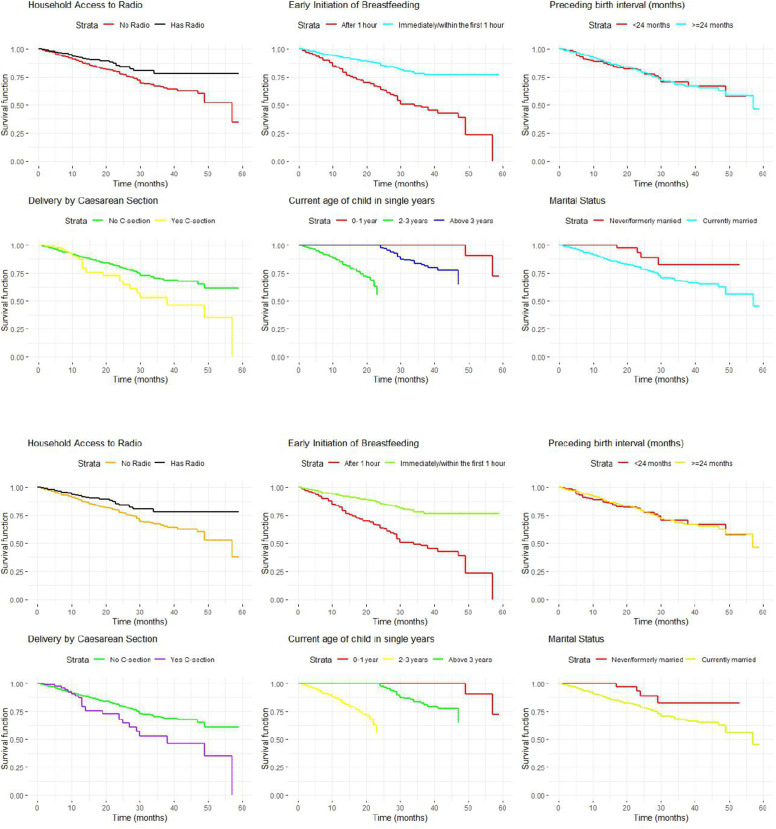
A. Comparison of Kaplan-Meier survival curves by the significant predictors of EBF in rural Ethiopia. B. Comparison of Nelson-Aalen survival curves by the significant predictors of EBF in rural Ethiopia.

Figs 2A and 2B above present the Kaplan-Meier and Nelson-Aalen survival curves indicating the probability of continuing EBF over time, stratified by the significant predictors like household access to radio, cesarean delivery, early initiation of breastfeeding, child’s age, preceding birth interval, and marital status. The survival curves for the household access to radio indicated that mothers from households without radio access exhibit a higher probability of continuing EBF for longer periods compared to mothers from households with radio access. The remaining survival curves are visualized in Figs 2A and 2B.

**Fig 3:**
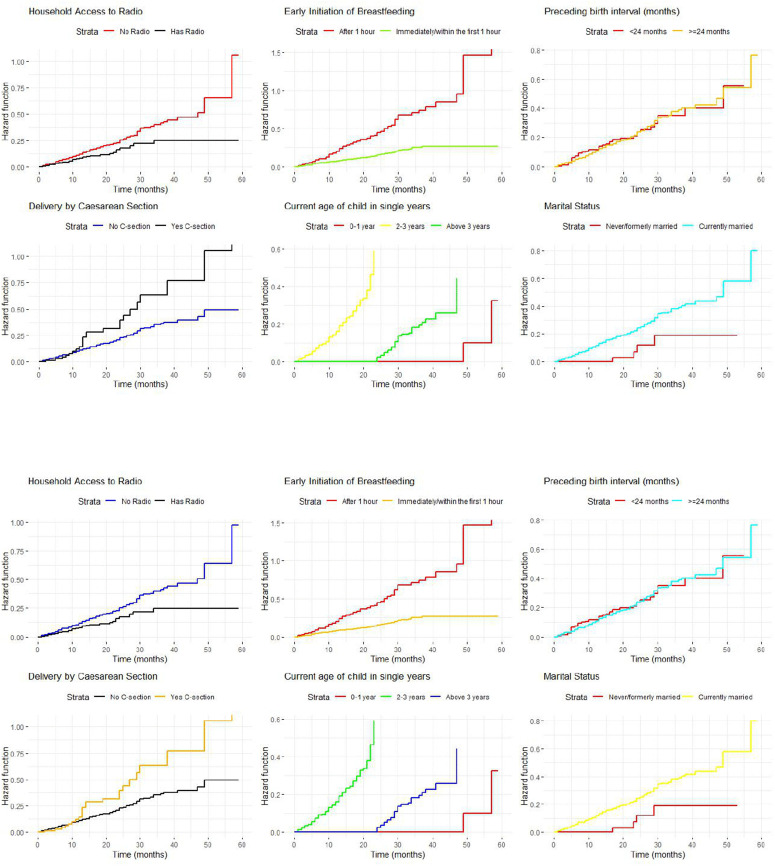
A. Comparison of Kaplan-Meier hazard curves by the significant predictors of EBF in rural Ethiopia. B. Comparison of Nelson-Aalen hazard curves by the significant predictors of EBF in rural Ethiopia.

Conversely, Figs 3A and 3B display the Kaplan-Meier and Nelson-Aalen hazard curves, indicating the probability of ceasing EBF over time stratified by the significant predictors. The hazard curves for the household access to radio indicated that mothers from households with radio access show a higher probability of ceasing EBF compared to their counterparts from households without radio access. The remaining hazard curves are displayed in Figs 3A and 3B.

### Multivariable analysis

Multivariable analysis was performed using the Cox proportional hazards model, along with exponential, Weibull, log-logistic, and log-normal parametric survival models, including all significant covariates identified in the univariable analysis with p-values from 0.2 to 0.25 or less, in addition to the preliminary steps of multivariable analysis at a 5% significance level. In Cox PH, Weibull, log-logistic and log-normal survival models, the significant predictors of the duration of exclusive breastfeeding in rural Ethiopia at the 5% level of significance included radio, cesarean delivery, region, early initiation of breastfeeding, current age of the child in years, birth interval, and marital status. However, in the exponential model, all of the aforementioned predictors except radio, and in the log-normal model, delivery place, were added to the aforementioned predictors and became significant predictors of exclusive breastfeeding in rural Ethiopia at the 5% level of significance (Table 4).

**Table 4:** Model comparison for the candidate survival time models.

Model	AIC	BIC	Log-likelihood
Cox	2732.435	2788.520	−1350.217
Exponential	2456.269	2542.591	−1212.135
Weibull	2352.591	2449.702	−1158.295
Log-logistic	2370.339	2467.451	−1167.17
Log-normal	2394.341	2496.849	−1178.171

Finally, the covariates in these survival time models that were taken into consideration in this investigation were used to compare the models. Of all candidate survival time models, the Weibull parametric survival model was the best-fitted model (Table 4).

**Fig 4:**
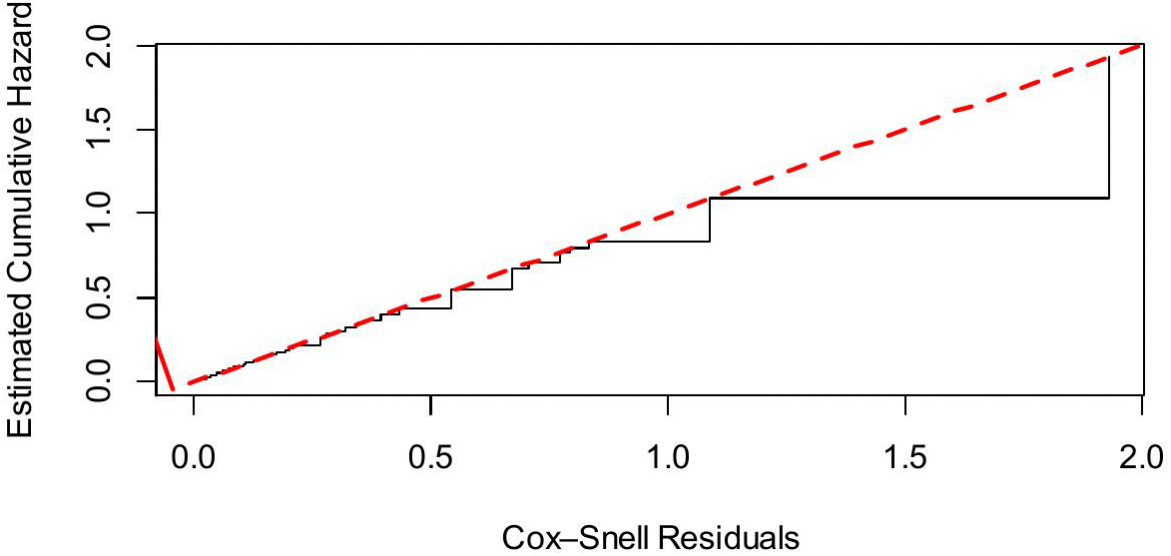
Cox-Snell residuals for a parametric Weibull model.

Finally, model adequacy checking was tested using the Cox-Snell residual plot, indicating that the predicted Nelson-Aalen cumulative hazard of the residuals closely approached the 45^0^ reference line. This suggests that the Weibull model showed an overall best-fit model to the EBF data, though minor deviations at higher residual values were observed due to censoring and few observations, which is expected (Fig 4).

### Predictors of the duration of exclusive breastfeeding in rural Ethiopia

From the final multivariable analysis of the Weibull parametric survival model displayed in Table 5 below, radio, cesarean delivery, region, early initiation of breastfeeding, child’s age, birth interval, and marital status were the significant predictors of exclusive breastfeeding in rural Ethiopia at the 5% level of significance. Mothers from households with a radio (HR = 1.274, p-value = 0.020; 95% CI: 1.039, 1.563) indicated that, holding the other covariates constant, mothers from households with radio access have a 27.4% higher risk of stopping exclusive breastfeeding earlier compared to those without radio access. Mothers who had a cesarean delivery (HR = 0.573, p-value = 0.001; 95% CI: 0.446–0.737) were 43% less likely to quit exclusive breastfeeding early than those who had a vaginal delivery.

**Table 5:** Predictors of exclusive breastfeeding in rural Ethiopia using the Weibull parametric survival model, EMDHS 2019 (n = 1628).

Predictors	$\hat{\beta }$	P-value	HR	95%CI for HR
Intercept	6.834	<0.001	929.259	[298.110, 2896.663]
Radio				
No	Ref.			
Yes	0.242	0.020	1.274	[1.039, 1.563]
Cesarean delivery				
No	Ref.			
Yes	−0.556	<0.001	0.573	[0.446, 0.737]
Region				
Tigray	Ref.			
Afar	−1.753	<0.001	0.173	[0.079, 0.381]
Amhara	−0.902	0.025	0.406	[0.184, 0.893]
Oromia	−0.970	0.017	0.379	[0.170, 0.843]
Somali	−2.188	<0.001	0.112	[0.051, 0.247]
Benishangul	−0.765	0.073	0.466	[0.202, 1.072]
SNNPR	−1.217	0.002	0.296	[0.135, 0.650]
Gambela	−1.256	0.002	0.285	[0.128, 0.633]
Harari	−1.512	<0.001	0.220	[0.097, 0.501]
Dire Dawa	−1.454	<0.001	0.234	[0.102, 0.536]
Early initiation of breastfeeding				
After 1 hour	Ref.			
Immediately/within the first 1 hour	0.441	<0.001	1.554	[1.341, 1.800]
Current age of child (in years)				
Above 3 years	Ref.			
0-1 year	−1.488	<0.001	0.226	[0.120, 0.425]
2-3 years	−0.505	0.123	0.604	[0.318, 1.147]
Birth interval				
<24 months	Ref.			
>=24 months	−0.200	0.034	0.819	[0.681, 0.985]
Marital status				
Never/Formerly married	Ref.			
Currently married	−0.666	0.016	0.514	[0.298, 0.884]

**NB:** Ref. = Reference Category, $\hat{\beta }\;$= Coefficient Value, HR = Hazard Ratio, 95% CI for HR = 95% Confidence Interval for Hazard Ratio.

The findings of this study also revealed that exclusive breastfeeding practices exhibit significant regional disparities throughout Ethiopia in rural communities. Compared to mothers in Tigray (reference category), those in Afar (HR = 0.173, p-value < 0.001, 95% CI: 0.079, 0.381), indicating an almost 83% lower risk of stopping EBF early; Amhara (HR = 0.406, p-value = 0.025, 95% CI: 0.184, 0.893), showing almost 59% lower risk to cease EBF; Oromia (HR = 0.379, p-value = 0.017; 95% CI: 0.170, 0.843), almost 62% lower risk of stopping EBF; Somali (HR = 0.112, p-value < 0.001, 95% CI: 0.051, 0.247), almost 89% lower risk of stopping EBF early; SNNPR (HR = 0.296, p-value = 0.002, 95% CI: 0.135, 0.650), almost 70% lower risk of stopping EBF; Gambela (HR = 0.285, p-value = 0.002, 95% CI: 0.128, 0.632), almost 71% lower risk of stopping EBF early; Harari (HR = 0.220, p-value < 0.001, 95% CI: 0.097, 0.501), almost 78% lower risk of stopping EBF; and Dire Dawa (HR = 0.234, p-value < 0.001, 95% CI: 0.102, 0.536), almost 77% lower risk of stopping EBF.

Mothers who initiated breastfeeding within the first hour had a 55.4% higher risk (hazard) of stopping EBF earlier compared to those who initiated after one hour (HR = 1.554, p-value < 0.001; 95% CI: 1.341–1.800). Several maternal and child-related factors significantly influence the duration of exclusive breastfeeding, including the child’s age, birth interval, and marital status. Younger infants are more likely to be exclusively breastfed, as shown by mothers with children aged 0–1 year having a 77.4% lower risk of stopping exclusive breastfeeding early (HR = 0.226, p-value < 0.001; 95% CI: 0.120, 0.425) compared to older infants. Birth interval also plays a significant role in exclusive breastfeeding duration. Mothers with a birth interval of 24 months or more (HR = 0.819, p-value = 0.034; 95% CI: 0.681, 0.985) were 18.1% less likely to stop exclusive breastfeeding early compared to those with shorter birth intervals. Breastfeeding practices are also influenced by marital status. Mothers who were currently married had a 49% lower risk of ending exclusive breastfeeding early than mothers who were never married or had previously been married (HR = 0.514, p-value = 0.016; 95% CI: 0.298, 0.884).

## Discussion

In the current study, survival time models were employed to identify the significant predictors of the duration of exclusive breastfeeding among infants fewer than six months in rural Ethiopia using the 2019 EMDHS dataset. According to the findings of time-to-event models, household access to radio, cesarean delivery, region, early initiation of breastfeeding, current age of the child, birth interval, and marital status were the significant predictors at the 5% level of significance. We discuss the general findings obtained below.

The findings of this study emphasize a significant public health issue concerning EBF in rural Ethiopia. Accordingly, a mere 15.1% of mothers residing in rural areas were not engaged in exclusive breastfeeding of their infants at the time the survey was conducted, which had already commenced the introduction of supplementary foods or liquids beyond breast milk. The lack of adherence to recommended breastfeeding protocols poses significant risks to infant health, including increased vulnerability to malnutrition, infections, and early childhood morbidity.

Furthermore, the investigation uncovered significant regional variations in EBF practices, indicating that geographic context is associated with considerable differences in maternal feeding behaviors. The Somali and Afar regions exhibited the highest percentages of infants who were not engaged in EBF, recorded at 4.2% and 2.6%, respectively. These pastoral and semi-arid regions may encounter distinct obstacles, including restricted access to maternal health education, entrenched cultural feeding customs, and insufficient healthcare infrastructure, which could impede compliance with exclusive breastfeeding recommendations. In contrast, regions like Benishangul demonstrated one of the lowest occurrences of non-EBF, quantified at 0.55%. These figures imply that localized initiatives, potentially encompassing community health extension programs or reinforced cultural norms favoring breastfeeding, might prove more efficacious in advancing EBF in these regions [4]. The considerable regional variation in EBF prevalence aligns with the findings of spatial and geospatial analyses conducted in Ethiopia, demonstrating that geographic location substantially affects breastfeeding practices [8,26]. These studies emphasized the significance of tailoring interventions to align with the cultural, infrastructural, and socioeconomic settings of individual regions, rather than adopting a uniform approach.

Mothers residing in households with radio access were 27.4% more likely to cease EBF early compared to those without radio access, indicating that radio ownership was significantly associated with shorter duration of EBF. This finding is in line with the previous research done by [2], who reported that mothers with access to mass media infant feeding counseling are more likely to practice EBF than those without such access. While media exposure is expected to promote appropriate infant feeding practices, radio exposure alone does not directly influence EBF cessation. This could be because households having radio access might differ in socioeconomic factors and exposure type to promote EBF, such as maternal employment, exposure to urban norms, or economic activities compared to their counterparts.

On the other hand, compared to vaginal birth, cesarean delivery was associated with a 43% reduced risk of early cessation of exclusive breastfeeding. This reduction may be attributed to improved postpartum care and counseling in clinical environments, which may support breastfeeding practices, consistent with previous research [10,15,26]. Comparing to the Tigray region, mothers residing in Afar, Amhara, Oromia, Somali, SNNPR, Gambela, Harari, and Dire Dawa regions exhibited significantly lower probabilities of cessation of EBF, with the Somali region demonstrating the most substantial protective effect, which corresponds to about 89% reduction in risk. These findings illustrate considerable differences in breastfeeding practices across regions, likely influenced by cultural traditions, accessibility to healthcare, or local health advocacy efforts, as also corroborated by the research conducted by [10,19].

Maternal delays in initiating breastfeeding beyond one hour post-delivery resulted in a 55.4% decreased likelihood of premature cessation of EBF. This finding supports existing literature indicating that early breastfeeding initiation within one hour fortifies the mother-infant relationship and promotes prolonged breastfeeding durations, which might increase the hazard of EBF as well [24,26]. Age of child also affected EBF, with neonates aged 0–1 year having a 77.4% lower probability of stopping EBF, which indicates EBF increased as the age of the infant increased, aligning with the previous research done by [16]. Mothers who had a birth spacing of 24 months and longer had an 18.1% reduced chance of ending EBF. This is likely because they enjoyed better recovery and could concentrate more on caring for their infants. Furthermore, women who are currently married were 49% less likely to stop EBF early compared to those who have never been married or those who were previously married, emphasizing the importance of support from partners due to Ethiopian people birth tradition and a stable home environment in maintaining healthy feeding practices, a conclusion that is supported by [25].

## Limitations of the study

Although this study offers useful insights into the factors that predict the duration of EBF in rural Ethiopia, the analysis was limited to the variables that were available in the 2019 EMDHS dataset exclusively. Consequently, some potentially significant psychological and cultural factors, like maternal mental health and spousal education and support, cultural beliefs, and attitude toward EBF were excluded due to secondary data constraints. Furthermore, the findings of this study may not be generalizable to urban environments or other countries with different healthcare systems and sociocultural frameworks, as the study was exclusively focused on rural Ethiopia. Moreover, the potential recall bias, which could lead to misclassification due to retrospective reporting; social desirability bias; and the cross-sectional design of the study introduce temporal ambiguity, are factors subject to influencing the outcomes under investigation.

## Conclusion

The purpose of this study was to investigate the predictors of the duration of EBF among infants under six months in rural Ethiopia, utilizing data from the 2019 EMDHS and applying survival time models. Hence, the findings of this study revealed that household access to radio, cesarean delivery, region, early initiation of breastfeeding, child’s age, preceding birth interval, and marital status were all statistically significant predictors of the duration of EBF in rural Ethiopia at the 5% level of significance. Specifically, exposure to radio increases the likelihood of mothers discontinuing EBF by 27.4%; conversely, cesarean section deliveries decrease the cessation of EBF by 43%. The research also identified significant regional variations in EBF, with mothers in Afar, Amhara, Oromia, Somali, SNNPR, Gambela, Harari, and Dire Dawa regions demonstrating a lower propensity to cease EBF, ranging from 59% to 89%.

Furthermore, initiating breastfeeding within the first hour post-delivery has considerable implications; mothers who postpone breastfeeding beyond one hour face a 55.4% higher risk of terminating EBF. The infant’s age additionally influences this dynamic; infants aged between 0 and 1 year show a 77.4% decreased probability of discontinuing breastfeeding. Longer birth intervals were associated with an 18.1% likelihood of early cessation of EBF, and marital status also affects early cessation of EBF; currently married women were 49% less likely to stop EBF prematurely. These findings emphasize the complex nature of EBF behavior and stress the necessity of region-specific, culturally attuned treatments that address both household and community-level factors. To improve EBF practices in rural Ethiopia, initiatives should focus on timely breastfeeding initiation, providing robust support to mothers through health communications, and addressing sociodemographic disparities to improve maternal and child health outcomes.

## Abbreviations

AIC: Akaike Information Criterion
BIC: Bayesian Information Criterion
EAs: Enumeration Areas
EBF: Exclusive Breastfeeding
EIBF: Early Initiation of Breastfeeding
DHS: Demographic and Health Surveys
EDHS: Ethiopia Demographic Health Survey
EMDHS: Ethiopia Mini Demographic Health Survey
EPHI: Ethiopian Public Health Institute
ICF: International Classification of Functioning
CSA: Central Statistical Agency
HR: Hazard Ratio
AHR: Adjusted Hazard Ratio
CI: Confidence Interval
PH: Proportional Hazards
Ref.: Reference Category
SNNPR: Southern Nations, Nationalities, and People’s Region
WHO: World Health Organization
